# Draft Genome Sequence of Butyricimonas faecihominis 30A1, Isolated from Feces of a Japanese Alzheimer’s Disease Patient

**DOI:** 10.1128/MRA.00462-19

**Published:** 2019-06-06

**Authors:** Tien Thi Thuy Nguyen, Kenshiro Oshima, Hidehiro Toh, Anushka Khasnobish, Yusuke Fujii, Kensuke Arakawa, Hidetoshi Morita

**Affiliations:** a Graduate School of Environmental and Life Science, Okayama University, Okayama, Japan; b College of Agriculture and Forestry, Hue University, Hue, Vietnam; c Foundation for Advancement of International Science, Ibaraki, Japan; d Medical Institute of Bioregulation, Kyushu University, Fukuoka, Japan; e Fundamental Laboratory, Ohayo Dairy Products Co., Ltd., Okayama, Japan; University of Rochester School of Medicine and Dentistry

## Abstract

Butyricimonas faecihominis 30A1, a butyrate-producing bacterium, was isolated from feces of a Japanese Alzheimer’s disease patient. Here, we report the draft genome sequence of this organism. This paper is the first published report of the genomic sequence of a *Butyricimonas* sp.

## Abstract (precis)

Butyricimonas faecihominis 30A1, a butyrate-producing bacterium, was isolated from feces of a Japanese Alzheimer’s disease patient. Here, we report the draft genome sequence of this organism.

## ANNOUNCEMENT

Alzheimer’s disease (AD) is a progressive neurodegenerative disorder of the central nervous system in the elderly population, characterized by an onset of dementia. Previous studies have suggested the link between alterations in the gut microbiota and the development of AD (1–3). Moreover, it has been reported that butyrate or butyrate-producing bacteria improved memory function in mouse models of dementia-related diseases, including AD (4, 5). Thus, we focused on butyrate-producing bacteria in the gut of AD patients. We have found and isolated Butyricimonas faecihominis 30A1 from feces of an 87-year-old Japanese AD patient (6). The genus *Butyricimonas* was first described in 2009 (7) and comprises four species (*B. faecihominis*, B. synergistica, B. paravirosa, and B. virosa) belonging to the family *Odoribacteraceae* within the phylum *Bacteroidetes* (7, 8). *Butyricimonas* spp. are obligate anaerobic, nonpigmented, Gram-negative rod, motile, and non-spore-forming bacteria. *B. faecihominis* was first isolated from human feces (8). Here, we report the draft genome sequence of *B. faecihominis* 30A1.

The 30A1 strain was isolated on blood liver (BL) agar for 2 to 4 days at 37°C in a Shel Lab Bactron anaerobic chamber (Sheldon Manufacturing) and was cultivated in Gifu anaerobic medium broth for 48 h under the same conditions. The genomic DNA of this strain was extracted with phenol-chloroform (9) and quantified using a NanoDrop 2000 spectrophotometer (Thermo Fisher Scientific). The double-stranded DNA concentration was measured using a Qubit fluorometer (Invitrogen). The genomic libraries were constructed using an Illumina Nextera XT DNA library prep kit and were paired-end sequenced on an Illumina MiSeq platform using the MiSeq reagent kit v2 (500 cycles), yielding 630,056 reads that provided 24.1-fold coverage of the genome. The resulting reads, after trimming low-quality bases from the 3′ end of the raw sequence reads, were assembled using Newbler v2.8 (Roche) with default parameters. The assembled genome consists of 808 scaffolds, with a total length of 4,752,022 bp and an *N*_50_ value of 11,636 bp. The assembled genome has a G+C content of 43.1% and contains 3,777 protein-coding genes predicted by Prokka v1.12 (10).

We have confirmed that the 30A1 strain was able to synthesize butyrate (6). A previous study has suggested that the *Bacteroidetes* phylum in the human gut can synthesize butyrate through four different pathways (11). We infer that the 30A1 strain can synthesize butyrate from acetoacetyl-coenzyme A (CoA) through crotonoyl-CoA using the *paaH*, *paaF*, *bcd*, *atoD*, *ptb*, and *buk* genes that were carried by the 30A1 genome. These genes were also found in the genomes of *B. virosa*, *B. synergistica*, and Odoribacter splanchnicus DSM 20712^T^ (12), which belongs to the same *Odoribacteraceae* family. In contrast, *Bacteroides* spp., which belong to the same *Bacteroidetes* phylum, do not carry all of these genes. Thus, the presence of these genes for butyrate synthesis may be one of the genomic characteristics of the *Odoribacteraceae* family. The genome information of this species will be useful for further studies of its physiology, taxonomy, and ecology.

### Data availability.

The draft genome sequence for *B. faecihominis* 30A1 has been deposited in the DDBJ/GenBank/EMBL databases under the accession numbers BHZJ02000001 to BHZJ02000808. The raw sequence data have been deposited in the Sequence Read Archive (accession number SRR8627645) and are available under BioProject accession number PRJNA524187.
